# Changes in trend of cerebral venous sinus thrombosis

**Published:** 2019-01-05

**Authors:** Banafsheh Shakibajahromi, Nahid Ashjazadeh, Anahid Safari, Afshin Borhani-Haghighi

**Affiliations:** 1 Clinical Neurology Research Center, Shiraz University of Medical Sciences, Shiraz, Iran; 2 Stem Cells Technology Research Center, Shiraz University of Medical Sciences, Shiraz, Iran

**Keywords:** Cerebral Veins, Sinus Thrombosis, Venous Thrombosis, Patient Outcome Assessment, Trends

Although cerebral venous sinus thrombosis (CVST) is less common than arterial stroke, it affects younger patients.^1^ Few studies have been performed to show the trend of CVST outcome over time.^2^ We aimed to assess the trend of CVST epidemiology and its outcome in Namazee hospital, Shiraz, Iran. This is a high volume referral center for cerebrovascular diseases in southern Iran.

Two retrospective studies were conducted on adult patients with definite diagnosis of CVST in two periods of time, 2000-2008^3^ and 2012-2016.^4^ We compared the demographic characteristics, risk factors and the outcome at the time of discharge between these two time periods. We applied Stata software (version 11, StataCorp LLC., College Station, TX, USA) for statistical analysis. Table 1 summarizes the results of comparison of CVST characteristics between the two time periods. 

**Table 1:** Comparison of CVST characteristics between time periods 2000-2008 and 2012-2016

**Variables**	**2000-2008 (n = 124)**	**2012-2016 (n = 174)**	**P**
**Demographic factors**	**Mean ± SD**	**Mean ± SD**	
Age (year)	34.01 ± 10.25	37.8 ± 11.2	0.003
	**n (%)**	**n (%)**	
Women	87 (70.2)	128 (73.6)	0.518
Risk Factors			
Female Related Causes*	67 (77.0)	96 (75.0)	0.691
OCP*	57 (65.5)	73 (57.0)	0.139
Pregnancy/ Post-partum*	10 (11.5)	23 (18.0)	0.124
Inflammatory/Rheumatologic diseases	13 (10.5)	5 (2.9)	0.007
Infection	11 (8.9)	14 (8.1)	0.806
Thrombophilia	9 (7.2)	42 (24.3)	< 0.001
Other (Trauma, Malignancy, …)	12 (9.7)	15 (8.6)	0.744
Idiopathic	16 (12.9)	27 (15.6)	0.514
Outcome			
Hospital mortality	18 (14.5)	9 (5.2)	0.006
Morbidity (mRS: 3-5)	44 (35.5)	30 (17.2)	< 0.001
Recurrent Thrombosis	12 (9.7)	11 (6.3)	0.278

SD: Standard deviation; CVST: Cerebral venous sinus thrombosis; OCP: Oral contraceptive pills; mRS: Modified Rankin Scale

* Percent is calculated among the women.

Morbidity and mortality of CVST in our region have diminished dramatically. This is most probably because of the advances in imaging techniques, which lead to more accurate and rapid diagnosis of CVST. Admission of patients with CVST in stroke units may also contribute to the decreased morbidity and mortality. Furthermore, better education of patients who are in risk of thrombosis about the possibility of developing CVST and its alarming symptoms in the recent years, has resulted in earlier admission of patients.

Female gender-related causes are still the most frequent risk factors. It is common that Iranian women misuse oral contraceptive pills (OCP) in order to delay their menstruation during religious ceremonies such as Haj and fasting month of Ramadan.^5^^,^^6^ Although not significant, OCP use has declined among our patients with CVST. The increasing frequency of thrombophilia can be attributed to more complete diagnostic work-ups in the recent years. The decrease in frequency of inflammatory diseases cannot be explained.

## Notes:

**How to cite this article:** Shakibajahromi B, Ashjazadeh N, Safari A, Borhani-Haghighi A. Changes in trend of cerebral venous sinus thrombosis. Iran J Neurol 2019; 18(1): 33-4.
